# Multidimensional prognostic indices for use in COPD patient care. A systematic review

**DOI:** 10.1186/1465-9921-12-151

**Published:** 2011-11-14

**Authors:** Wouter D van Dijk, Lisette van den Bemt, Saskia van den Haak-Rongen, Erik Bischoff, Chris van Weel, Johannes CCM in 't Veen, Tjard RJ Schermer

**Affiliations:** 1 Radboud University Nijmegen Medical Centre, Department of Primary and Community Care, Nijmegen, the Netherlands; 2 QUARTZ Integrated Care Support Service, Helmond, the Netherlands; 3 Sint Franciscus Gasthuis, Department of Chest Diseases, Rotterdam, the Netherlands

**Keywords:** COPD, index, multidimensional, prognosis

## Abstract

**Background:**

A growing number of prognostic indices for chronic obstructive pulmonary disease (COPD) is developed for clinical use. Our aim is to identify, summarize and compare all published prognostic COPD indices, and to discuss their performance, usefulness and implementation in daily practice.

**Methods:**

We performed a systematic literature search in both Pubmed and Embase up to September 2010. Selection criteria included primary publications of indices developed for stable COPD patients, that predict future outcome by a multidimensional scoring system, developed for and validated with COPD patients only. Two reviewers independently assessed the index quality using a structured screening form for systematically scoring prognostic studies.

**Results:**

Of 7,028 articles screened, 13 studies comprising 15 indices were included. Only 1 index had been explored for its application in daily practice. We observed 21 different predictors and 7 prognostic outcomes, the latter reflecting mortality, hospitalization and exacerbation. Consistent strong predictors were FEV_1 _percentage predicted, age and dyspnoea. The quality of the studies underlying the indices varied between fairly poor and good. Statistical methods to assess the predictive abilities of the indices were heterogenic. They generally revealed moderate to good discrimination, when measured. Limitations: We focused on prognostic indices for stable disease only and, inevitably, quality judgment was prone to subjectivity.

**Conclusions:**

We identified 15 prognostic COPD indices. Although the prognostic performance of some of the indices has been validated, they all lack sufficient evidence for implementation. Whether or not the use of prognostic indices improves COPD disease management or patients' health is currently unknown; impact studies are required to establish this.

## Background

Chronic obstructive pulmonary disease (COPD) is a chronic respiratory condition, with a high impact on patients' wellbeing, health care utilization, and mortality [1]. The (progressive) airflow obstruction that is characteristic for COPD is closely related to its morbidity and mortality [1-3]. Hence, degree of airflow obstruction is generally considered to be the key factor for staging COPD severity and to guide and monitor treatment [1]. However, (pharmacological) interventions to both stabilize the progression of airflow obstruction and reduce premature mortality are disappointing [4-6]. Currently, there is a growing recognition that COPD should no longer be regarded as a synonym for airflow obstruction alone, but instead as a multidimensional condition that comprises several phenotypes [1,7-10]. In addition, patients tend to die from other diseases than COPD [11]. Therefore, severity staging and treatment of COPD should follow this view and take into account the heterogenic nature of the disease.

Apart from airflow obstruction, a range of other factors have been identified as important predictors of future risk on morbidity and mortality in patients with COPD, including smoking [12,13], degree of dyspnoea [14], age [15], exercise capacity [16], body mass index [17], exacerbations [18], (cardiovascular) comorbidity [19], and quality of life [20]. Combining several of these prognostic factors in a multidimensional index embodies the current holistic vision on COPD, and may ultimately provide physicians with a powerful tool to assess and monitor disease severity in order to guide decision making and improve patient outcome [9,21]. Such an index clearly needs to meet several prerequisites in terms of accuracy, predictive and discriminative power, internal and external validity and, last but not least, practicability of use in different health care settings [22,23]. In addition to monitoring and guiding decision making in patients, a prognostic index could also be used to predict patients' health care utilization, to identify and target particular high-risk groups within the COPD patient population, or for risk stratification in clinical trials.

With the growing number of prognostic COPD indices developed for clinical use, an overview to facilitate the discussion of the pros and cons of using prognostic indices in COPD patient care is warranted. In this paper, we aim to identify, summarize and compare all published multidimensional prognostic indices that assess and stage disease severity in patients with COPD. Apart from providing insight in their performance, we aim to present the effects of their application in daily patient care, that will be important to help clinicians, scientists and health policy makers in deciding whether or not to implement an index.

## Methods

We performed a systematic literature review on multidimensional prognostic indices for COPD.

### Literature search

We comprehensively traced indices by systematically searching the Pubmed and Embase literature databases (see Additional File 1 and 2 for search strategies). We conducted the Pubmed search first and included all records published before 27 September 2010. The Pubmed search was not restricted to language. As the first index traced in Pubmed was published in 2004, the subsequent Embase search was limited to records published after 2002, and to records in English, German, French or Dutch. Two authors (LvdB and TS) independently screened the titles and abstracts of the retrieved records to identify potentially eligible studies, and next assessed eligibility by full-text assessment. Disagreement was resolved by consensus, and if necessary by a third party (SvdH). In addition, we searched the bibliographies of included articles for relevant cross-references and searched Pubmed and Embase for index-related articles reporting supplementary information on validity, usefulness, and/or additional prognostic outcomes.

### Study selection

We included primary publications of prognostic indices that: 1) were developed specifically for COPD; 2) were developed and validated in samples with COPD patients only; 3) were developed for assessment of COPD patients with stable disease (i.e., not during exacerbations or hospitalizations); 4) predict future outcome of COPD; 5) consist of more than one component; 6) have a scoring system for their components. As the quality of the prognostic indices was part of our evaluation we did not exclude indices based on poor quality.

### Data extraction and quality assessment

The following data were extracted from each included study: age, gender, and airflow obstruction as measured by FEV_1_/FVC (forced expiratory volume in one second/forced vital capacity), FEV_1_% (FEV_1 _as percentage of the predicted value), and index purposes, predictors, prognostic outcomes and prognostic value. Ideally, a prognostic index progresses through three consecutive stages: development, validation, and impact quantification. There is no consensus on the 'best' method for development. Indices can be build from a set of predefined predictors that may either be established predictors and/or be selected based on practicability of measuring the predictor. The predictors could be selected based on their availability and pragmatism or by statistical modeling. The subsequent performance and validity of the developed index should be measured by its discriminative properties: how well does the index distinguish patients with poor prognosis from patients with good prognosis (c-statistic 0.50 equals random guessing, > 0.70 is good performance, 1.0 is maximum), and by its accuracy (how well do predictions from the index measures up to the real observations, i.e. 'goodness-of-fit'). Since not all studies that report prognostic indices for COPD included this information in their methods, we additionally extracted data on correlation, multiple regression and survival analyses. To conclude the validation stage, an index should be investigated in a separate validation cohort in order to test generalization and, if applicable, it should be compared with conventional prognostic measures (in the case of COPD with FEV_1_%. In general, if based on statistical selection of predictors and in smaller samples, the index is likely to perform better in its primary cohort. Before implementing an index in daily practice, the final challenge is to assess the effects on patient outcome and health care by impact studies [21-23].

WvD and LvdB independently scored the methodological quality of the studies using a structured screening form (Additional File 3), that exposes potential systematic bias and the validity of the generated indices: from good to poor, based on the average score of 6 items. This form was derived from a framework specifically developed for the evaluation of the methodological quality of prognosis studies in systematic reviews by Hayden et al, who extracted and grouped all quality items from 163 prognostic reviews [24].

## Results

### Study selection

**Figure 1:**
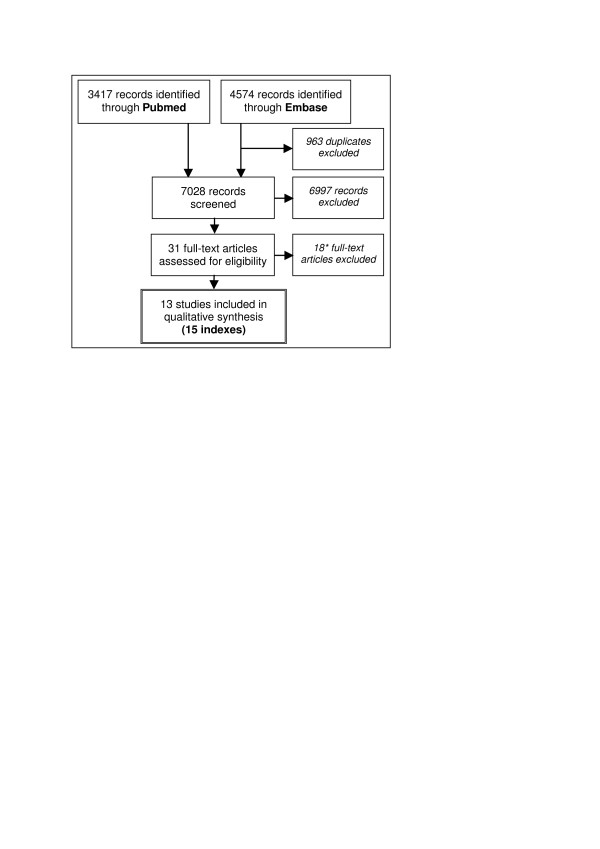
**PRISMA Flow Diagram**. * Three articles excluded based on lack of scoring system, 11 due to absence of prognostic outcome, 3 were not the original publication of the index, and 1 was not COPD specific.

Figure 1 summarizes the selection of studies. Of the 7,028 identified records, 31 were assessed for eligibility. After full-text assessment, we excluded 18 articles because of a lack of a scoring system for individual patients [25-27], absence of prognostic outcome [28-38], not being the original publication of an index [39-41], and not being COPD specific [42]. Consequently, we included 13 studies that altogether reported 15 multidimensional COPD indices (Table 1 and Additional Files 3 and 4). The first index was published in 2004 [43], whereas 2009 yields most studies. The prior purpose of most studies was to develop or improve an index for clinical use. Only 1 index was initially developed for epidemiologic use [30]. Index modeling was based on either achieving maximal predictive value or on practicability and mostly used statistical selection of predictors.

**Table 1:** Essential index summaries: general information, predictive ability, population and study quality.

INDEX	GENERAL INDEX INFORMATION				INDEX QUALITY				POPULATION			STUDY QUALITY			KEY *STRENGTHS*/FLAWS
Scale and publication year	Index aim	Cited (SCI)	Predictors	Outcome	**Predictor ind. Sig**.	Discrimi nation	Accu racy	Compare to	N	Age (year)	Mean FEV_1_%	Score	Model building	**Val**. cohort	
**ADO** 10-points scale 2009	C	14	Age Dyspnoea (MRC or GCRQ) Obstruction (FEV_1_%)	Death	Yes	Modest	Good	BODE	232 342	72 68	52% all < 80%	Fairly good	A*	+	*Accurate* **Age is paradoxical** **Elderly patients only**
**BODE** 10-point scale 4 categories 2004	C	580	BMI (length/weight^2^) Obstruction (FEV_1_%) Dyspnoea (MRC score) Exercise tolerance (6MWD)	Death Respiratory death	Yes	*Death*: Good *Respiratory:* no report	-	FEV_1_%	207 625	66 67	39-47%	Good	A*	+	*Good quality* *Good discrimination* **Severe COPD only** **Exclusion of CVD**
➣ ***BODEx*** *9-point scale* *4 categories* *2009*	*D*	*7*	*BMI (length/weight^2^)* *Obstruction (*FEV_1_*%)* *Dyspnoea (MRC score)* *Exacerbations*	*Death*	*All but 1*	*Good*	*-*	*BODE*	*185*	*71*	*48%*	*Fair*	*P*	*-*	*Good discrimination* **Elderly males only**
➣ ***e-BODE*** *12-point scale* *4 categories* *2009*	*D*	*7*	*Exacerbations* *BMI (length/weight^2^)* *Obstruction (*FEV_1_*%)* *Dyspnoea (MRC score)* *Exercise tolerance (6MWD)*	*Death*	*All but 1*	*Good*	*-*	*BODE*	*185*	*71*	*48%*	*Fair*	*A*	*-*	*Good discrimination* **Elderly males only**
➣ ***mBODE*** *10-point scale* *2007*	*D*	*3*	*BMI (length/weight^2^)* *Obstruction (*FEV_1_*%)* *Dyspnoea (MRC score)* *Exercise max. O_2_-use*	*Correlation BODE*	*-*	*-*	*-*	*BODE*	*50*	*63*	*63%*	*Fair*	*A*	*-*	**Small sample** **Restricted outcome and analysis**
**COPDSS: COPD Severity Score** 35-point scale 2008	E	7	Respiratory symptoms Systemic corticosteroids Other COPD medications Hospitalization/Intubation Home oxygen use	Respiratory outpatient Respiratory ED visit Respiratory hospital	- (index yes)	-	-	-	267	65	54%	Fair	P	±^$^	*Nomogram* **Diagnosis based on self-report** **No outcome confirmation**
**CPI: COPD Prognostic Index** 100-point scale 3 categories 2008	C	2	Quality of life (SGRQ/CRQ) Obstruction (FEV_1_%) Age Gender BMI History of ED/exacerbation History of CVD	Death Hospitalization Exacerbation	Depends on outcome	*Model*: Good *Val.cohort*: no report	-	-	5856 2946	64 64	44%	Fairly poor	P*	+	*Adequate statistics* *Large sample* **Selective reporting** **Pooled analysis**
**DOREMI BOX** 10-point scale 2 categories 2008	D	?	Dyspnoea (ATS) Obstruction (FEV_1_%) Rate of Exacerbation Movement (6MWD) BMI (length/weight^2^) Blood OXygen (PaO_2_)	Correlation BODE Death	Yes (2 no report)	-	-	BODE	84	59	35% (18-73%)	Fairly good	A	-	*Clear descriptions* *Long follow-up* **Small sample** **Limited statistics** **Severe population**
**DOSE**, 8-point scale 2009	C	3	Dyspnoea (MRC score) Obstruction (FEV_1_%) Smoking Exacerbations	Correlation BODE Exacerbation Hospitalization for exacerbation	- (index partly)	*Hospital* Good *Exacerbation* no report	-	BODE	375 81 133	69 73 67	42-67% all < 80%	Fairly poor	P*	+^#^	**Difficult, complex and selective reporting** **Violated own protocol**
**HADO** 12-point scale 3 categories 2006	C	5	Health (new questionnaire) Activity (new questionnaire) Dyspnoea (Fletcher) Obstruction (FEV_1_%)	Death	No	Modest	-	FEV_1_%	611	67	50% all < 80%	Fairly good	P	-	*Clear descriptions* *Compared to FEV_1_%* **Modest discrimination** **Predictors debatable**
**Niewoehner (1)** 422-point scale 2007	C	27	Age Obstruction (FEV_1_%) Hospitalization COPD duration Productive cough Antibiotics Systemic corticosteroids Theophylline	Exacerbation	Yes	Modest	Seems good^@^	-	1829	69	36% all < 60%	Fair	A*	-	*Large sample* **No validation cohort** **Severe COPD/males only** **No outcome confirmation**
**Niewoehner (2)** 249-point scale 2007	C	27	Age Obstruction (FEV_1_%) Hospitalization Unscheduled visits Cardiovascular disease Oral corticosteroids	Hospitalization for exacerbation	Yes	Good	Seems good^@^	-	1829	69	36% all < 60%	Fair	A*	-	*Good discrimination* *Large sample* **No validation cohort** **Severe COPD/males only** **No outcome confirmation** **Predictor is outcome**
**PILE **2010 10-point scale 4 categories	C	0	Obstruction (FEV_1_%) Interleukin-6 Knee extensor strength	Death	Yes	Good	-	FEV_1_% mBODE	268	73	63% all > 30%	Fair	A*	-	*Long follow-up* *Good statistics* **No validation cohort**
**SAFE **2007 9-point scale 4 categories	C	5	SGRQ score (questionnaire) Air-flow limitation (FEV_1_%) Exercise tolerance (6MWD)	Exacerbation (correlation)	-	-	-	-	86	68	43% (12-98%)	Fair	P	-	**Small sample** **Poor statistics**
**Schembri** **(TARDIS)** 16-point scale 2009	C	0	Age BMI Dyspnoea (MRC score) Obstruction (FEV_1_%) Hospitalization Influenza vaccination	Hospitalization for COPD or respiratory death as 1 outcome	Yes	-	-	-	3343	?	? all < 80%	Fair to fairly poor	A*	-	*Large sample* **No validation cohort** **Composite outcome** **Limited statistics**

Indices are sorted alphabetically. Index aim: primary purpose of utilization: C) clinical use, D) further development of existing index, E) epidemiologic use; Score: average of bias screening form; Model building: priorities in model development (* if predictors are statistically selected): A) accuracy, P) pragmatism; Val. cohort: separate validation cohort; SCI: science citation index; Predictor ind. sig.: independent significance of predictors for outcome; $: same cohort, different time-window; #: Selective use and reporting of cohorts;^@^: reliability plot without statistics. CVD: cardiovascular disease

### Index descriptions

The indices were established from study samples that ranged from 50 to 8802 patients, whereas their mean follow-up ranged from 0.5 to 6.1 years. The weighted average of FEV_1_% was 44% (study means range 35 to 63%). We observed large variation in predictors, scoring systems and outcomes (Table 1 and Additional File 4). From all indices, 21 predictors (Table 2) emerged for 7 different outcomes (Table 3). Across indices, shared predictors were often measured with different instruments, variously categorized, and diversely valued and weighted. For instance, 4 instruments were used to measure dyspnoea and 6 categorizations were applied to stage FEV_1_% (Additional File 4). Two of these categories deviated from the guidelines they referred to [43,44], i.e. the cut-point of 65% was not mentioned in those guidelines [45,46]. Altogether, the indices included 3 to 8 predictors and their maximum scoring ranged from 8 to 422 points. Airflow obstruction - expressed as FEV_1_% - was included in all indices except for one [47]. Other frequently included predictors were age, dyspnoea, exercise tolerance and Body Mass Index (BMI).

**Table 2:** COPD outcome predictors (n = 21), grouped by disease components.

COMPONENT	PREDICTOR	N
**Physiologic**	- *Obstruction (FEV_1 _as % from predicted)*	14
	- *Blood oxygen (PaO2)*	1
	- *Exercise maximum oxygen consumption*	1
**Biomarkers**	- *Interleukin-6*	1
**Physical**	- *BMI (length/weight2)*	7
	- *Exercise tolerance (6MWD)*	4
	- *Knee extensor strength*	1
**Medical history**	- *COPD duration*	1
	- *(Rate of) exacerbation (unscheduled visits, emergency department visits, hospitalisation, intubation)*	8
	- *History of cardiovascular disease*	2
**Demographics**	- *Age*	5
	- *Gender*	1
	- *Smoking status*	1
	- *Influenza vaccination status*	1
**Signs & symptoms**	- *Dyspnoea (ATS, MRC score, GCRQ, and Fletcher)*	9
	- *Quality of life (SGRQ)*	2
	- *Health status (CRQ and ad hoc questionnaires)*	2
	- *Respiratory symptoms like cough*	2
**Treatment**	- *COPD medication (including steroids and theophylline)*	3
	- *Antibiotics*	1
	- *Home oxygen*	1

N is number of indices.

**Table 3:** Summary of different outcomes used in prognostic COPD indexes.

OUTCOME	SPECIFIC INDEX OUTCOME	N
**Death**	- *All-cause death*	*8*
	- *Respiratory death*	2*
**Hospitalisation**	- *All-cause hospitalization*	*1*
	- *Respiratory hospitalization*	4*
**Exacerbation**	- *Unscheduled respiratory outpatient visit*	*1*
	- *Respiratory emergency department visit*	*1*
	- *Any exacerbation*	*4*

N is number of studies. * One study used these outcomes as a composite outcome

Mortality, as defined by all-cause or respiratory cause, was the most frequent predicted outcome (n = 8) [15,43,44,48-51], followed by all-cause or respiratory hospitalization (n = 4) [47,48,52,53], exacerbation (n = 4) [48,52-54], and unscheduled in- and outpatient visits (n = 1) [47]. Correlation with another index [43] was the only outcome in one study [55]. One study combined respiratory death and hospitalization as a composite outcome [56]. Two studies also analyzed the change of the index over time and the association with outcome [47,52]. Definitions and methods of measurement of shared outcomes varied across indices, particularly for exacerbations (Additional File 3).

### Performance and quality

Table 1 reveals the statistical methods used to validate the performance of the respective indices, which were heterogenic and often deficient. Only 3 indices were compared to FEV_1_% and showed a modest, not formally tested, improvement of discriminative power in contrast to FEV_1_% (c-statistics increased from 0.63 - 0.65 to 0.68 - 0.74). Only 5 studies used some sort of validation cohort. Apart from these primary studies, only 2 indices had been additionally validated for their outcomes in succeeding studies [43,55], whereas 3 indices had been additionally evaluated for other outcomes (Additional File 4) [43,48,55]. Remarkably, we retrieved only 1 (non-controlled pilot) impact study that implemented a multidimensional prognostic index in COPD patient care (11 patients included, 6 months follow-up, no significant improvements due to case management directed by the index) [57].

The overall methodological quality varied from fairly poor to good (Table 1). All studies described the baseline characteristics of their sample adequately, but only half described their process of patient recruitment and selection. Nine studies did not properly report the study attrition, information on drop-outs in particular, and one study appeared to violate its own protocol for predictor selection [52]. Measurements sometimes remained undefined and were inadequate for several indices, in particular with respect to (lack of) outcome confirmation.

## Discussion

We identified 15 different multidimensional prognostic COPD indices, of which several have been validated appropriately. Although most indices were developed for clinical use, as yet, these indices lack impact studies to demonstrate effects on patient outcome and health care when implemented in daily patient care. The indices may improve population-based prediction of the natural course of COPD compared to looking at airway obstruction (as measured by FEV_1_%) alone, in terms of mortality, hospitalizations and exacerbations, although discrimination still remains modest. The diversity in populations, (the weighting of) predictors and (the definition of) outcome, hampers any overall recommendations on which index to prefer for predicting prognosis in patients with COPD. However, our overview of the indices currently available can guide future research in selecting the most suitable index/indices for impact studies.

### Strengths and limitations

Our systematic search in two leading medical literature databases limited the chance of missing an index. The Embase search did not reveal additional indices. As there is no consensus on how to perform quality assessment of studies in systematic reviews of prognostic studies, we based our study assessments on Hayden's previously reported criteria, in an effort to enhance (the validity of) our study evaluations [24]. A limitation of our review is the (inevitable) subjectivity when judging the methodological quality of the studies underlying the indices, including deficient blinding of the studies. We attempted to counteract subjectivity by independent and systematic scoring by two investigators. Finally, we focused on prognostic indices for stable disease only and excluded indices that had been developed for acute exacerbations, or that assessed current disease severity only without predicting future outcome(s).

### Interpretation

Although the quality of the studies generally appeared fair, the lack of proper analyses on their predictive performance and the lack of uniformity across indices hampers any recommendations regarding preference for any particular index. Altogether, rather than truly improving predictive abilities, the large number of COPD indices mainly substantiate the important value of airflow obstruction on population level. Although regarded as a relatively difficult and impractical measurement to obtain, this single predictor is required for the diagnosis of COPD [1]. Most other predictors would be relatively easy to perform, except for time and space consuming exercise tests, of which age and dyspnoea in particular seem reliable and predictive measures.

As smoking is an important, easily measured and modifiable predictor of COPD prognosis, we were surprised to observe that of 6 studies that reported smoking, only 1 (fairly poor) study included smoking in its index. Seven studies did not have information about smoking available. Obviously, smoking status and smoking intensity may change repeatedly over time and self-reported reliability is poor, requiring confirmation tests like urine analyses. Furthermore, age is an excellent predictor in both cardiovascular and COPD indices [58]. However, age seems a difficult predictor to value in a prognostic index for secondary prevention, such as with COPD [59]. Age is non-modifiable and its usefulness in tailoring individual COPD treatment accordingly is less obvious: it may prohibit treatment of young patients, and younger patients would ironically have less urgency to quit smoking.

Although the COPD indices could be used for epidemiologic and research purposes, they would ideally support (cost-effective) decision making on individual treatment in order to improve outcome in patients with COPD [9,21], or even as to provide individual prognoses. So far, only one (pilot) study actually implemented a prognostic COPD index in patient care, without showing significant improvements in health (care) [57]. As the practical abilities of COPD indices remain unexposed, they fail to tackle the current urge to improve treatment programs [7,9,60].

Ongoing research by prospective follow-up of a wide range of (known and new) predictors specifically aims to further improve both phenotyping and the prognostic capacity of COPD indices [61,62]. However, as the 'proof of the pudding' would be to actually study the effect of applying such an index in directing decision making and outcome in individual patients, research priorities would need to shift towards these objectives, preferably by means of impact studies [9,21,23]. Different indices might prove to be suitable for different populations, purposes and settings [63]. Although impact studies have not (yet) been used commonly for prognostic indices [23], we believe that COPD treatments with variable effects further urge to study the effects of implementing an index in daily practice.

### Recommendations

Following the development and validation of the current indices, the next step for COPD researchers is to perform impact studies, instead of developing more indices [23]. These studies should establish both the applicability and the impact on health(care) of implementation of these indices in daily clinical practice. An impact study requires a randomized controlled trial that quantifies the effect of using a prognostic index on predefined outcomes including decision making, patient outcome, and cost effectiveness, compared with usual care without implementing the index [23].

Another issue is whether or not a prognostic index should integrate predictors, preferably modifiable [9], of which treatments can indeed improve the index scores and outcome [64,65]. Ultimately, indices should be integrated with predictive, preventive, personalized and participatory (P4) medicine [66], i.e. index-tailored (self)management strategies for individual COPD phenotypes. To improve applicability in clinical practice, a 5^th ^P could be added for 'practical'.

Finally, applying a prognostic index in a patient population other than the one in which it was developed may require recalibration or modification. For example, Puhan et al. recently reported improved prognostic performance of the BODE index after population-based recalibration [15]. Age-specific calibration or stratification of an index allows adjustments that can resolve the dilemma of this strong but troublesome predictor [58]. Apart from age, calibration to critically-ill patients in need of (decisions on their) ICU admission and/or ventilation, might prove its benefit. In addition, this population could also benefit from non-modifiable predictors that merely define a high-risk group of patients [67].

## Conclusions

We identified 15 multidimensional prognostic indices specifically developed for COPD patients. Although the overall prognostic performance appears moderate to good and several indices have been validated, there currently is a lack of evidence that prognostic indices improve decision making, treatment or outcome in patients with COPD. The next challenge is to perform impact studies that determine if implementation of specific indices can indeed improve individual health(care) in specific settings.

## Competing interests

There are no conflicts of interest of any kind related to this paper. Conflicts of interest, not related to this paper: Johannes in 't Veen received fees for consultancy or lectures from Astra Zeneca, Boehringer Ingelheim, Cheisi, Novartis and Nycomed. Tjard Schermer received fees or grants for his department used for research, education, equipment, salaries, etc. from NutsOhra Fund, GlaxoSmithKline, AstraZeneca and Boehringer Ingelheim. Chris van Weel received unrestricted fees or grants for his department for research, education, equipment, salaries, etc. from Bayer, NovoNordisk, Astra Zeneca, Boehringer Ingelheim, GlaxoSmithKline and Novartis. Wouter van Dijk, Lisette van den Bemt, Erik Bischoff and Saskia van den Haak-Rongen do not have any conflict of interest.

## Authors' contributions

All authors read and approved the final manuscript. WvD: Acquisition and interpretation of data, drafting of the manuscript. LvdB: Study concept and design, acquisition and interpretation of data, drafting of the manuscript. SvdH: Drafting of the manuscript. EB: Drafting of the manuscript. CvW: Drafting and critical revision of the manuscript. JiV: Drafting and critical revision of the manuscript. TS: Study concept, design and supervision, acquisition and interpretation of data, drafting and critical revision of the manuscript

## Supplementary Material

Additional file 1 **Pubmed Database Search: **Pubmed Search strategy. Click here for file

Additional file 2 **Embase Database Search: **Embase Search strategy. Click here for file

Additional file 3 **Index Quality Assessments: **Contains the quality assessment form and all index quality assessments. Click here for file

Additional file 4 **Detailed Index Summaries: **Contains a more extensive summary of the index characteristics and properties. Click here for file
