# Supplementary material for: Multidimensional prognostic indices for use in COPD patient care. A systematic review
Source: Respir Res. 2011 Nov 14;12(1):151. doi: 10.1186/1465-9921-12-151 (PMC3228786; doi:10.1186/1465-9921-12-151)
Supplement: Additional file 1 — Pubmed Database Search: Pubmed Search strategy. [file 1465-9921-12-151-S1.DOC]

**Additional file 1**

**Pubmed database Search**

Search ((("pulmonary emphysema"[MeSH Terms] OR ("pulmonary"[tiab] AND "emphysema"[tiab]) OR "pulmonary emphysema"[tiab] OR "pulmonary disease, chronic obstructive"[MeSH Terms] OR ("pulmonary"[tiab] AND "chronic"[tiab] AND "obstructive"[tiab]) OR "copd"[tiab] OR "chronic bronchitis"[tiab])) AND (("Health Status Indicators"[Mesh] OR "Predictive Value of Tests"[MeSH]) OR (Health Status[tiab] OR (Severity[tiab] AND (index[tiab] OR indices[tiab])) OR (multidimensional[tiab] OR staging[tiab] OR grading[tiab] OR (Predictiv*[tiab] AND model*[tiab]))))) NOT (Editorial[ptyp] OR Letter[ptyp] OR Practice Guideline[ptyp] OR Addresses[ptyp] OR Bibliography[ptyp] OR Biography[ptyp] OR Case Reports[ptyp] OR Clinical Conference[ptyp] OR Comment[ptyp] OR Consensus Development Conference[ptyp] OR Consensus Development Conference, NIH[ptyp] OR Corrected and Republished Article[ptyp] OR Dictionary[ptyp] OR Directory[ptyp] OR Duplicate Publication[ptyp] OR Guideline[ptyp] OR Historical Article[ptyp] OR Interactive Tutorial[ptyp] OR Interview[ptyp] OR In Vitro[ptyp] OR Lectures[ptyp] OR Legal Cases[ptyp] OR Legislation[ptyp] OR News[ptyp] OR Newspaper Article[ptyp] OR Patient Education Handout[ptyp] OR Periodical Index[ptyp] OR Portraits[ptyp] OR Published Erratum[ptyp] OR Retracted Publication[ptyp] OR Retraction of Publication[ptyp] OR Scientific Integrity Review[ptyp] OR Technical Report[ptyp] OR Twin Study[ptyp] OR Webcasts[ptyp])
