# Supplementary material for: Multidimensional prognostic indices for use in COPD patient care. A systematic review
Source: Respir Res. 2011 Nov 14;12(1):151. doi: 10.1186/1465-9921-12-151 (PMC3228786; doi:10.1186/1465-9921-12-151)
Supplement: Additional file 2 — Embase Database Search: Embase Search strategy. [file 1465-9921-12-151-S2.DOC]

**Additional file 2**

**Embase database search**

1. obstructive airway disease/ or airway constriction/ or airway obstruction/ or bronchitis/ or bronchus obstruction/ or bronchus stenosis/ or chronic obstructive lung disease/ or lung emphysema/

2. (obstructive airway disease or airway constriction or airway obstruction or bronchitis or bronchus obstruction or bronchus stenosis or chronic obstructive lung disease or lung emphysema or pulmonary emphysema or chronic obstructive pulmonary disease or copd).mp. [mp=title, abstract, subject headings, heading word, drug trade name, original title, device manufacturer, drug manufacturer]

3. 1 or 2

4. classification/ or clinical classification/ or disease classification/ or "international classification of impairments, disabilities and handicaps"/ or nursing classification/ or staging/

5. scoring system/

6. disease severity/ or chronic disease/ or deterioration/ or disability severity/ or prognosis/

7. prediction/

8. health status/ or fitness/ or functional status/ or health disparity/ or physical mobility/

9. rating scale/

10. (classification or staging or scoring or prognosis or prediction or index or rating scale).mp. [mp=title, abstract, subject headings, heading word, drug trade name, original title, device manufacturer, drug manufacturer]

11. 4 or 5 or 7 or 9 or 10

12. (deterioration or severity or health status or fitness or functional status or health disparity or risk or survival or physical mobility).mp. [mp=title, abstract, subject headings, heading word, drug trade name, original title, device manufacturer, drug manufacturer]

13. 6 or 8 or 12

14. 3 and 11 and 13

15. limit 14 to (dutch or english or french or german)

16. limit 15 to yr="2003 - 2010"
