# Supplementary material for: Multidimensional prognostic indices for use in COPD patient care. A systematic review
Source: Respir Res. 2011 Nov 14;12(1):151. doi: 10.1186/1465-9921-12-151 (PMC3228786; doi:10.1186/1465-9921-12-151)
Supplement: Additional file 3 — Index Quality Assessments: Contains the quality assessment form and all index quality assessments. [file 1465-9921-12-151-S3.DOC]

**Additional file 3**

**INDEX QUALITY ASSESSMENTS**

**Quality assessment form** Page 2

**Quality assessments:**  Page 3-15

- ADO Page 3
- BODE Page 4
- COPDSS Page 5
- CPI Page 6
- DOREMI Page 7
- DOSE Page 8
- E-BODE and BODEx Page 9
- HADO Page 10
- mBODE Page 11
- Niewoehner Page 12
- PILE Page 13
- SAFE Page 14
- TARDIS Page 15

Form to assess quality in prognostic studies on the basis of framework of potential biases, derived from Hayden JA. [[1]](#footnote-2)

| *Potential Bias* | *Items To Be Considered for Assessment of Potential Opportunity for Bias* |
| --- | --- |
| **Study participation**  The study sample represents the population of interest on key characteristics, sufficient to limit potential bias to the results. | - The source population or population of interest is adequately described for key characteristics. - The sampling frame and recruitment are adequately described, possibly including methods to identify the sample (number and type used, e.g., referral patterns in health care), period of recruitment, and place of recruitment (setting and geographic location) - Inclusion and exclusion criteria are adequately described (e.g., including explicit diagnostic criteria or “zero time” description). - There is adequate participation in the study by eligible individuals. - The baseline study sample (i.e., individuals entering the study) is adequately described for key characteristics. |
| **Study attrition**  Loss to follow-up (from sample to study population) is not associated with key characteristics (i.e., the study data adequately represent the sample), sufficient to limit potential bias. | - Response rate (i.e., proportion of study sample completing the study and providing outcome data) is adequate. - Attempts to collect information on participants who dropped out of the study are described. - Reasons for loss to follow-up are provided. - Participants lost to follow-up are adequately described for key characteristics. - There are no important differences between key characteristics and outcomes in participants who completed the study and those who did not. |
| **Prognostic factor measurement**  The prognostic factor of interest is adequately measured in study  participants to sufficiently limit potential bias. | - A clear definition or description of the prognostic factor measured is provided (e.g., including dose, level, duration of exposure, and clear specification of the method of measurement). - Continuous variables are reported or appropriate (i.e., not data-dependent) cut-points are used. - The prognostic factor measure and method are adequately valid and reliable to limit misclassification bias (e.g., may include relevant outside sources of information on measurement properties, also characteristics, such as blind measurement and limited reliance on recall). - Adequate proportion of the study sample has complete data for prognostic factors. - The method and setting of measurement are the same for all study participants. - Appropriate methods are used if imputation is used for missing prognostic factor data. |
| **Outcome measurement**  The outcome of interest is adequately measured in study participants to sufficiently limit potential bias. | - A clear definition of the outcome of interest is provided, including duration of follow-up and level and extent of the outcome construct. - The outcome measure and method used are adequately valid and reliable to limit misclassification bias (e.g., may include relevant outside sources of information on measurement properties, also characteristics, such as blind measurement and confirmation of outcome with valid and reliable test). - The method and setting of measurement are the same for all study participants. |
| **Confounding measurement and account**  If any relevant and practical confounders are possible, are these accounted for and does the model hold up? | - Important potential confounders, including treatments (key variables in conceptual model), are measured reliably and valid and have clear definitions. - The method and setting of confounding measurement are the same for all study participants and appropriate methods are used if imputation is used. - Important potential confounders are accounted for in the study design (e.g., matching for key variables, stratification, or initial assembly of comparable groups). - Important potential confounders are accounted for in the analysis (i.e., appropriate adjustment). |
| **Analysis**  The statistical analysis is appropriate for the design of the study, limiting potential for presentation of invalid results. | - There is sufficient presentation of data to assess the adequacy of the analysis. - The strategy for model building (i.e., inclusion of variables) is appropriate and is based on a conceptual framework or model. - The selected model is adequate for the design of the study. - There is no selective reporting of results. |

**ADO:**

**Study Participation: Fairly Good**

Poor: - Source population key characteristics nor primary participation rate are not shown in this paper

Fair: - Participation 100% from previous study

Good: - Sampling is from elderly rehabilitation or admitted exacerbations cohort.

- Recruitment 2004-2006, 1 Swiss and 9 Spanish hospitals, based on spirometry and some exclusion criteria

- Baseline sample is described well for key characteristics

- Spanish participants do not have differences with those who declined.

- Swiss: severe FEV1 45%

- Spanish: moderate FEV1 52%

**Study Attrition: Fairly Poor**

Poor: - Response rate not described

- Loss to follow-up and their characteristics not described

Good: - Attempts to collect information (particularly on outcome) of drop-outs by 5 telephone calls, hospital visit, general practitioner contact or hospital record

**Prognostic Factor Measurement: Fair**

Poor: - Cut-points of FEV1 and age appear arbitrarily

- Proportion of complete data not described

Fair: - Clear definition although partly description of the factor measurements and their validity

- Setting differs between cohorts and within Spanish cohort. Method of dyspnoea measurement differs, although it is exchangeable

Good: - Imputation by the means of 50 datasets for Age, BMI, Dyspnoea and 6MWD

**Outcome Measurement: Good**

Good: - Clear definition; all cause (date of) death, follow-up > 30 months

- Blind measurement by contacting patient, partner, GP or hospital record

- Confirmation when deceased by GP or hospital record

**Confounding Measurement and Account: Fair/Unsure**

- There are several possible confounders measured like gender, packyears, cardiovascular disease, PaO2 and medication which are not accounted for in the paper. These are all unlikely to influence the validity of the index but may influence the goodness-of-fit and discriminative power.

**Analysis: Fairly Good**

Fair: - Strategy is somehow artificial: replacing one factor of BODE by another preset factor.

- Recalibration of the intercept per cohort is debatable but might prove useful.

Good: - Sufficient data

- Fractional polynomial analyses by bootstraps to define the significant factors

- 2 cohorts for model building and validating

- Hosmer-Lemeshow and c-statistic to describe the usefulness of the index.

**BODE:**

**Study Participation: Fair**

Poor: - Source and participation is not described.

- Poor description of recruitment: Who? Where? How?

- Sample has low FEV1, many packyears and much exclusion based on comorbidity.

Good: - Good description of selection and baseline characteristics.

**Study Attrition: Good**

Poor: - No reasons for drop-out.

Good: - Loss to follow-up low (4%) and same (not described in detail) baseline characteristics.

- Drop-outs and their families have been approached.

**Prognostic Factor Measurement: Fair**

Poor: - Only small group deceased in model building

- BODE index cut-points and weighting is not argumented

- No description of how measurmeents were performed and by whom

- No information on missing data and/or imputation

Good: - Prognostic factors described extensively

- Cut-points based on other studies.

**Outcome Measurement: Good**

Good: - Follow-up described well (>2 years, each 3-6 months).

- Outcome: (respiratory) death by medical record and death certificate as collected by investigator on site.

**Confounding Measurement and Account: Fair**

Poor: - Age and hematocrite were not included as confounder, although they were statistical significant predictors.

Good: - Adjusted for Charlson index as confounder

**Analysis: Fairly Good**

Poor: - The data of forward regression to select the predictors is not presented.

Fair: - The analyses only reveal c-statistics on any death, not on respiratory death.

Good: - Model based on statistics, other studies and practicability

- Much data and appropriate analyses

- Statistical weighting of BODE predictors did not affect prediction.

**COPDSS:**

**Study Participation: Fair**

Poor: - Diagnosis is based on self report

Fair: - Source population is reasonably described in different paper (Eisner 2005), whereas baseline sample is reasonably described in this paper.

- Selection criteria are based on age, interview completion and diagnosis (Trupin 2003)

- Participation 53% from overall eligible patients at prescreening (age, interview) and 70% of final eligible individuals for validation sample.

Good: - Sampling frame and recruitment is at random by telephone number.

**Study Attrition: Fairly Good**

Fair: - Response rate is 76% the first year and 65% the second year.

- Drop-outs are not thoroughly described nor the attempts to collect their information.

Good: - It is claimed that subjects lost to follow-up are similar to those who complete the study

- 15% drop-outs are described: death

- Probability-of-attrition weighted analyses did not change results substantively.

**Prognostic Factor Measurement: Fair**

Poor: - Relies on recall

- Factors and weighting were based on reasoning only

Fair: - Continuous variables are reported for antibiotic use and dyspnoea, although their cut-points appear arbitrarily.

- Proportion with complete data is not described, although in a previous paper the original eligible patients reveal 0-2% missing values per item with appropriate imputations.

Good: - Clear definition of prognostic factor measured.

- Alternative weighting by factor analyses correlated closely (not shown)

- Factor measurement is by telephone interview by a professional interview firm

**Outcome Measurement: Fairly Poor**

Poor: - Outcome is based on recall, without confirmation.

- Outpatient visit cut-point is data dependent

Good: - Outcome is clearly described as 3 different respiratory-specific visits: outpatient, ED and hospitalization, measured yearly by telephone for all patients 1-3 years.

**Confounding Measurement and Account: Fair**

Fair: - Adjustments for available covariates (age, race, ...) for odds ratio and nomogram, although they are included in the C-statisitic

**Analysis: Fair**

Poor: - The analyses lack (description of) factor significance for the initial predicting model

- The first year is not included in the analyses, whereas the second year samples from this population

Fair - There is a reasonable amount of data

- Model building is based on reasoning and no statistics are involved except for alternative weighting. The cohort itself is only used for (concurrent) validation of different outcomes. (this paper, Eisner, Trupin)

- Internal validation by different time interval

- The index model is added to the confounders model to prove its validity.

Good: - (adjusted) odds ratios with significance are described for the prognostic index as well as for its change over time.

- Logistic regression for the initial predicting model.

- Nomogram to reveal the index value.

**CPI:**

**Study Participation: Fairly Poor**

Poor: - Source population, sampling frame, recruitment, selection criteria and hence participation rate are not described due to the study design.

Fair: - Selective population with severe COPD (FEV1% is 44%) and much CVD comorbidity (45%).

- 12 different previous studies, all treatment trials.

Good: - Baseline study sample is adequately described for key characteristics

**Study Attrition: Fair**

Poor: - No description of loss-to-follow and dropouts and hence the response rate or differences with completed results.

**Prognostic Factor Measurement: Poor**

Poor: - Cut-points are reported for BMI, Age, FEV1% and QOL, though seem data dependent.

- Cut-point for prognostic index tertiles are not shown or described.

- Clear description of QOL measurement, but nothing on other factors or their measurements (results copied from the previous studies).

Good: - Only 6,5% of data needed imputation

- Subset regression for imputations

**Outcome Measurement: Fair**

Poor: - Measurement method and setting are not described.

Good: - Description of outcome - death, hospitalization, exacerbation (acute respiratory episode requiring antibiotics or oral corticosteroids) and composite - including follow-up, based on reports of individual studies.

**Confounding Measurement and Account: Fairly Good**

Fair: - Whether predictors or confounders are selected from many factors based on significance and relevance. Therefore, possible confounders are not adjusted for but included in the model.

Good: - Adjustments for treatment effects of individual studies by stratification.

**Analysis: Fair**

Poor: - An overall C-statistic or ROC-curve of the validation group and hazard ratio of the composite model and validation group is lacking. The final index of the entire population lacks any statistics.

- Equations are not shown

Fair: - Almost sufficient presentation of data

- After analyses of the first two cohorts, they used the total cohort for their final index.

Good: - Backward stepwise combined with relevance of factor for model building, although based on availability of factors instead of selection prior to the studies.

- 2 different cohorts for model building and validation, although from the same population, they introduced a systematic time bias to increase validity.

- Adequate models: Cox regression, overall C-statistic, chi-square and negative binomial analyses.

**DOREMI BOX:**

**Study Participation: Fair**

Poor: - Source is not described

- Sampling frame and recruitment is not described

- Participation is not described

Good: - Inclusion and exclusion criteria are adequately described. Although inclusion criteria are limited and exclusion criteria are extensive

- Baseline key characteristics are described adequately

**Study Attrition: Fairly Poor**

Poor: - There is no information collected for drop-outs nor are there any reasons provided.

Fair: - Key characteristic for the baseline group as well as for the group completing the study. Differences not described.

Good: - Response rate 68/84

**Prognostic Factor Measurement: Fairly Good**

Poor: - Missing values and imputation are not described, therefore the proportion with complete data is unclear.

- Setting is not described

Fair: - Index cut-point is rather arbitrarily.

Good: - There is a clear and extensive description of prognostic measurement, which components are based on studies confirming an independent relation with outcome. All components are reliable and valid although exacerbation measurement is not mentioned.

- Cut-points are based on literature, although some are pragmatically combined.

**Outcome Measurement: Fairly Good**

Poor: - No clear confirmation particularly if outcome is negative.

Fair: - It is not described who measures (and confirms) outcome and when this is done.

Good: - Clear definition of outcome (death) and

- Follow-up: 36 months

- Outcome (death) is obtained from 2 Bulgarian registers and similar to all.

**Confounding Measurement and Account: Good**

Good: - Age and smoking packyears are studied for their potential bias, which appeared insignificant.

**Analysis: Fairly Good**

Poor: - No ROC-curve or correlation of model with outcome

- 6MWD and Dyspnoea not shown in t-test!

Fair: - No deaths after 3 years?

- BODE Hazard Ratio falls out of confidence interval by Celli due to?

- Only 1 cohort for both modelling as validating. Model building by literature and pragmatism without statistics. NB based on improvement of existing index.

Good: - Sufficient data

- Component analyses: t-test for outcome, correlation for concurrent validity.

- Cox for outcome and correlation with BODE.

**DOSE:**

**Study Participation: Fair/Unsure**

Poor: - No references of secondary data sources

- Participants are unclear: participation rate is lacking, and excluded patients of Devon data set are included in the participation number.

- Source population is scarcely described with scarce information on recruitment and selection criteria

Fair: - Sampling frames are partly described: part primary care, part respiratory clinic.

Good: - Clear goal for implementation and reason for selection of population

- Key characteristics of baseline sample are described with a FEV1 ranging from 42-67% predicted.

**Study Attrition: Poor/Unsure**

Poor: - Numbers are not clear and incorrect, although attrition seems mediate in the London cohort (107/133)

- Drop-outs are briefly described in one data set (London) as died or withdrawn, without any other description of information collection and characteristics.

**Prognostic Factor Measurement: Fair**

Poor: - Poorly reasoned selection of prognostic factors which appears contradictory to their aforementioned protocol.

Fair: - Prognostic measurement is reasonably described in the model building cohort only

- Missing data is excluded pairwise in model building. Nothing on validation studies.

- Proportion of study sample with complete prognostic data, when interpretable, ranges from 70-95%.

- Methods and settings were different, although this is mentioned and results are analysed separately

Good: - Cut points are based on literature and guidelines.

**Outcome Measurement: Poor**

Poor: - Outcome is compared with health status measured by SGRQ, which is not a health status instrument

- Many different cohorts, with many cohort dependent outcomes, with only selective reporting.

- 5 cohorts with only one used as prognostic validation, with as it appears includes 1 year of retrospective and 1 year prospective data.

- Prognostic outcome measurement is not clearly described (any confirmation?), nor mean follow-up.

- London study: 149 patients have outcomes, although only 133 patients have a DOSE score.

Fair: - Prognostic value ends up as insignificant or as small effect only (exacerbations, which is also an index factor).

- Only one of the three prognostic outcomes is defined (exacerbation London: seen by a physician with at least 2 consecutive days of increased respiratory symptoms of which 1 was a major symptom).

**Confounding Measurement and Account: Unsure**

- Whether not corrected for or not collected

**Analysis: Fairly Poor**

Poor: - There appears to be a very selective inclusion of variables in the model building as well as in the reporting of results, which depend on cohort and outcome.

- Unclear numbers and tests

- One outcome is the same as an index factor, exacerbations

- Validation by the Devon cohort is by a retrospective measure which is indirectly part of the index

- Goodness-of-fit and figures on DOSE score and hospitalisation/exacerbation are lacking.

- Wrong analyses for index change over time

- No DOSE-BODE correlation for Holland data

- Figure 2 suggests a strong relation, although numbers above DOSE score 5 are scarce.

Fair: - Model is developed for a different outcome than the validation outcome.

- Weighting was based on explained variance, however by wrong (parametric) analysis.

- Poisson regression for hospitalisations and exacerbations, although test is not for non-parametric distributions

Good: - R2 for index and health status

- Sufficient presentation of data to assess the adequacy of the analyses (which is not)

- Model building and prognostic validation by separate cohort AUC for future hospitalisation only (is it really future?)

- Spearman’s correlation DOSE-BODE in Japan

**e-BODE & BODEx:**

**Study Participation: Fairly Good**

Poor: - Poor description of recruitment setting (outpatients) and none of participation rate.

- 100% men!

Good: - Adequate description of recruitment period, selection criteria and baseline characteristics

**Study Attrition: Fairly Poor**

Poor: - Lost to follow-up not further described

Good: - 10% lost to follow-up in the 5 year period

**Prognostic Factor Measurement: Fair**

Poor: - No information on proportion with complete data or imputations

- No information on measurement settings

Fair: - Scarce arguments for cut-points of new predictor

Good: - Clear definition and description of all predictors with adequate measurements

**Outcome Measurement: Poor**

Poor: - Outcome not described

- Measurement not described

- Follow-up expectations not clearly described: a mean follow-up of 36 months does not match with 90% study attrition at 5 years.

**Confounding Measurement and Account: Good**

Good: - Confounders are looked at and analysed, and accounted for in the model.

**Analysis: Fairly Good**

Poor: - Only adjusted Cox analysis for final model

- No validation cohort

Unsure: - ROC and c-statistics only when >5 years follow-up

Good: - Sufficient data

- Univariate Cox survival for components and confounders

- Kaplan-Meier for survival curves

- ROC curves and c-statistics, comparisons of different curves.

**HADO:**

**Study Participation: Fairly Good**

Poor: - Unknown participation number.

Fair: - Source population is roughly described.

Good: - Adequate and extensive description of sampling frame, recruitment and selection criteria with adequate description of key characteristics.

**Study Attrition: Fairly Good**

Poor: - Attempts to collect information on drop-outs are at the most scarcely mentioned implicitly.

- No reason for their loss is provided.

Good: - Response rate is 585 of 611. 26 are lost to follow-up.

- Reasonable description of participants lost to follow-up, and as it appears they mostly have no differences with the other participants.

**Prognostic Factor Measurement: Fair**

Poor: - 2 prognostic factors are not tested before and their validity remains doubtful. However, an attempt is done to compare with other valid health measures. (partly successful)

- No proportion with complete data, nor imputation is mentioned.

- Obstruction cut-points appear data-dependent or incorrect.

- The index cut-points are not reasoned.

Good: - A clear definition of all prognostic factors is provided, with support from literature.

- Appropriate cut-points are used.

- Method and setting are similar to all participants.

**Outcome Measurement: Good**

Good: - A clear definition of the outcome is provided (mortality, 3 years) with a clear and valid measurement (visit + phone with confirmation by family, GP, medical records and/or mortality registries), similar to all participants

**Confounding Measurement and Account: Good**

Good: - Adjustment for possible confounders by first testing their influence on the model in an univariate analysis (not shown), and later inclusion of the significant ones in the multivariate analyses (reported)

**Analysis: Fair**

Poor: - Index numbers in table 4 and dyspnoea HR in table 3 are incorrect

- No weighting of index

Fair: - Model building was based on pragmatism beforehand and hence only one study group exists.

- Several models are selected. The most important ones however fail to reveal good evidence for the index (Cox and multivariate linear regression for the index component contributions).

Good: - Sufficient presentation of data

- There appears no selective reporting of results

- C-statistic for predicting mortality compared to FEV1%

**mBODE:**

**Study Participation: Fairly Poor**

Poor: - Source, recruitment and participation not described

Fair: - Selection criteria only described as by stale COPD as per GOLD criteria.

Good: - Sample size calculation

- Baseline sample adequately described

**Study Attrition: Unsure**

Poor: - Nothing described

**Prognostic Factor Measurement: Fairly Good**

Poor: - Setting not described

- Missing values, imputations and proportion with valid data not described.

Good: - Cut-points based on literature

- Same method for everyone

**Outcome Measurement: Fairly Good**

Poor: - Setting not described

Good: - BODE index as outcome implies the same comments as for the prognostic factor measurement (except for 6MWD, which appears the same and valid for all participants)

**Confounding Measurement and Account: -**

Not applicable

**Analysis: Good**

Good: - Sufficient data presented

- Model was based on adjustment of an existing index, based on results of previous studies.

- Model adequate for the design

**Niewoehner:**

**Study Participation: Fairly Good**

Poor: - Source population is not described for key characteristics

Good: - Adequate description of sample frame, recruitment and characteristics (2001-2003, All patients at 26 veteran affairs medical facilities at US.

- Extensive selection criteria including diagnostic criteria, although very selective (FEV1% 36%, male 99%, no never smokers, much CVD even though unstable/severe CVD is excluded)

- Adequate participation of eligible patients 80%

**Study Attrition: Fairly Poor**

Poor: - Participants loss-to-follow up are not described nor the attempts to collect information, except for COPD as an important reason of participants to quit the study drug

Good: - Response rate is about 85%

**Prognostic Factor Measurement: Fair**

Poor: - Cut-points seem data dependent

- The measurement and methods depend mostly on recall by questionnaire.

- The proportion with complete data on

- Continuous variables are not mentioned explicitly, although spirometry might be 15% according to previous paper.

Fair: - Definition of prognostic factors is not described, but only implicitly mentioned. For instance, it is unclear if productive cough needs to be chronic.

- Imputation is mentioned for one variable (longitudinal data analysis for spirometry).

Good: - Spirometry is according to ATS standards.

- Measurements seem the same for all

- The proportion with complete data on categorised data appears 99%.

**Outcome Measurement: Fairly Good**

Poor: - Exacerbations are not confirmed

- One outcome is part of the other outcome

Good: - A clear definition for outcomes hospitalization for exacerbation and exacerbation (at least 2 new or increased respiratory symptoms (cough, sputum, wheeze, dyspnoea, chest tightness) for at least 3 days and requiring hospital, antibiotics or systemic corticosteroids) are shown.

- The same measurements methods by monthly telephone calls and 3-monthly visits, gaining information based on case report forms and patient diaries, confirming hospitalisations by discharge summaries and medical records.

**Confounding Measurement and Account: Fair**

Poor: - Variables related to physical (experienced) health status and quality of life are missing.

Good: - Of a wide range of variables, all available variables are included in the model if they prove statistically significant .

**Analysis: Fair**

Poor: - There is no validation group

- Outcome is part of the index as a retrospective measure

- One outcome is part of the other outcome, while their indexes are different

Good: - Sufficient and complete presentation of data (except for statistics on the calibration plots)

- Strategy of model building by stepwise analysis after initial univariate analysis

- Cox regression for hazard ratios, followed by a c-index, risk nomogram and calibration plots.

- Bootstrapping for internal validity

**PILE:**

**Study Participation: Fairly Good**

Poor: - Source data recruitment strategies and participation rate not clear

Good: - Reasonably described source population with adequate information on selection criteria.

- Baseline sample selection criteria and characteristics adequately described, although smoke characteristic as % seems oddly constructed.

**Study Attrition: Fairly Poor**

Poor: - No further information on lost to follow-up

Good: - Response rate is adequate (96%), although minimal follow-up time is not clear: 5 years?

**Prognostic Factor Measurement: Fair**

Poor: - No standard reversibility test for spirometry

- Knee extensor only right side

- Cut-points knee extensor and Il-6 based on?

Fair: - Different cut-points men/women for knee extensor

Good: - Clear definition of prognostic factors and mostly appropriate measurements of all covariates

- Reasonable imputations for knee extensor (12%) and Il-6 (7%) described: adequate proportion complete data

- Appropriate cut-points FEV1

**Outcome Measurement: Good**

Good: - Clear definition

- Mortality by 6 months telephone calls and in-person visits, confirmed by hospital records, death certificates and informants.

**Confounding Measurement and Account: Fairly Good**

Good: - Adjustment of all available potential confounders, although confounders as comorbidity are sometimes measured by irreliable self-report and medication checks only

**Analysis: Fair**

Poor: - No arguments for weighting

- No validation cohort

Good: - Sufficient data and adequate strategy and design

- Censoring of lost to follow-up

- Covariates explored for associations (students t, chi-square, Kaplan-meier, Cox

- Adjusted HR for components

- Selection of predictors by stepwise regression

- Final Cox model with confounder adjustments

-Internal bootstrapping for evaluation model

-Robustness test by removing 10% highest FER

-AIC and BIC for model fits

-C-statistics for PILE, mBODE and FEV1%

**SAFE:**

**Study Participation: Fairly Good**

Poor: - Source population and participation not described

Good: - Adequate description of recruitment (place, setting, frame) and selection.

- Baseline study sample is adequately described, although not very representative.

**Study Attrition: Poor**

Poor: - Proportion completing the study, dropout handling, loss to follow-up and their characterization are not described.

**Prognostic Factor Measurement: Fair**

Poor: - Reversibility measurement is not clear.

- Missing values, imputations and proportion with complete data is not described.

Good: - Clear definition and description of prognostic factor and measurement (self-report and spirometry).

- Continuous variables are well described with clear cut-points.

- Same measurements for all, although it is not clear who performs the measurements

**Outcome Measurement: Fairly Good**

Fair: - Not clear who, where, and when is measured (recall or diary?).

Good: - Clear definition of outcome (exacerbation based on antibiotics or systemic corticosteroids for increased COPD symptoms), which is reasonably valid and reliably measured (self-report and review of medical records)

**Confounding Measurement and Account: Unsure**

Unsure: - Age could be a confounder, which could be but is not adjusted for.

**Analysis: Fairly Poor**

Poor: - FEV1% was not significant and not correlated with other components but was still included.

- FEV1% was not displayed in the k-means table.

- First model building, next correlation with prognostic outcome, but validation was within same cohort by internal reliability consistency only.

- A ROC-curve or c-statistic would be nice.

Fair: - Cut-points in model building were derived from K-means, except airflow obstruction

- Why quartile cut-points?

Good: - Sufficient data for assessment

**TARDIS:**

**Study Participation: Fairly Good**

Poor: - Source population not described for key characteristics

Fair: - Selection criteria are only shown by diagnostic criteria

Good: - Sampling frame and recruitment within GP setting at 1st COPD diagnosis by spirometry confirmation by nurse, between 2001 and 2005 in Scottish region

- About 50% participation

- Baseline key characteristics are well described

**Study Attrition: Poor**

Poor: - Not described, perhaps due to no follow-up criterion.

**Prognostic Factor Measurement: Fair**

Poor: - All continuous variables are categorised, but cut-points are not justified

Fair: - Prognostic factors are described very roughly without little details on background or exact measurement. For instance, measurements for housebound participants (4%) might be different but is not described.

Good: - Proportion with complete data appears implicitly around 95%

- Predictors with many missing values are excluded, so are patients with missing values of significant predictors

**Outcome Measurement: Fair**

Poor: - Both different outcomes are measured and analysed as one outcome

- Outcome is also part of the index as a predictor

Fair: - Follow-up for outcome is not mentioned

Good: - A clear definition by whether hospital admission or death based on COPD by direct patient interview, confirmed by morbidity records and mortality registries, similar to all patients.

**Confounding Measurement and Account: Fair**

Good: - Many variables are measured and tested for their significance as a predictor

**Analysis: Fair**

Poor: - The reported range of the index appears wrong (figure) and the index proportional hazard is missing.

- Goodness-of-fit and whether c-statistic or correlation between predicted and observed outcome is missing

- There is no validation or validation group.

Fair: - Almost sufficient presentation of data: non-significant predicting variables are not shown

- Weighting is derived from individual hazard ratios, although the strategy is dubious and as it appears from the hazard results incorrect

Good: - Backward elimination of all available covariates for model building

- Weibull proportional hazards and cumulative risk vs index score as models for index relevance

1. Hayden JA, Cote P, Bombardier C. Evaluation of the Quality of Prognosis Studies in Systematic Reviews. Ann Intern Med 2006;144:427-437. [↑](#footnote-ref-2)
