# Supplementary material for: Multidimensional prognostic indices for use in COPD patient care. A systematic review
Source: Respir Res. 2011 Nov 14;12(1):151. doi: 10.1186/1465-9921-12-151 (PMC3228786; doi:10.1186/1465-9921-12-151)
Supplement: Additional file 4 — Detailed Index Summaries: Contains a more extensive summary of the index characteristics and properties. [file 1465-9921-12-151-S4.DOC]

|  | **Study population** |  | **Quality criteria** |  |  |  | **Additional literature** | | |
| --- | --- | --- | --- | --- | --- | --- | --- | --- | --- |
| **Index** | **Patient number/follow-up/**  **setting** | **Population/selection bias** | **Objective in index development and model building** | **Study design** | **Validity** | **Quality:**  **Hayden** | **Cited**  **(SCI)** | **Val. studies** | **Alternative outcomes studied** |
| **ADO**  2009 | *Model*: 232 patients  *Validation*: 342 patients  Follow-up >30 months  Swiss (rehab.), Spain (inpatient) | *Model*: Old patients FEV_1_%: 45%  *Validation*: Post-admission (stable assessed), men↑, FEV_1_%: 52% All <80% | Thorough modeling of few predictors to obtain a well calibrated prognostic index, applicable in primary care and useful to manage treatment. | Prospective observational cohorts (model/validation) | By different cohort, after recalibration: Similar results. | Fairly good | 14 | - | - |
| **BODE**  2004 | *Model*: 207 patients  *Validation*: 625 patients  Median follow-up 28 months  resp clinic: Spain, Venezuela, US | FEV_1_%: 39-47%. Range?  >20 packyears  Exclusion co-morbidity (unstable/severe CVD) | Thorough modeling of many predictors to categorize and better predict outcome than FEV_1_: “improve comprehensibility in evaluation of COPD patients, and practical for widespread applicability | Prospective observational cohorts  (model/validation) | By different cohort: significant.  Similar results in different populations by country. | Good | 580 | 8 ^1^ ^2^ ^3^ ^4^ ^5^ ^6^ ^7^ ^8^ | Hospital ^9^ SGRQ^10^  Exacerb. ^11^ ^12^ ^13^  Death by longitudin. change ^14^ ^15^ |
| - **BODEx**   2009 | *185 patients. Mean follow-up 3 years Setting: outpatients* | *100% male. FEV_1_%: 48%*  *All smoke history* | *Simplify, improve, and test exacerbation as independent predictor from BODE.* | *Prospective observational cohort* | *Not clear* | *Fair* | *7* | *-* | *-* |
| - **e-BODE**   2009 | *185 patients. Mean follow-up 3 years Setting: outpatients* | *100% male. FEV_1_%: 48%*  *All smoke history* | *Simplify, improve, and test exacerbation as independent predictor from BODE.* | *Prospective observational cohort* | *Not clear* | *Fair* | *7* | *-* | *-* |
| - **mBODE**   2007 | *50 patients, cross-sectional*  *Setting: ?primary care?, Brazil.* | *Moderate: FEV_1_%: 63%. Range?* | *Preliminary modification of BODE, to strengthen the usefulness of BODE* | *Cross-sectional cohort* | *Not shown* | *Fair* | *3* | *1 ^16^* | *Any death ^17^* |
| **CPI: COPD Prognostic Index**  2008 | *Model*: 5856 patients  *Validation*: 2946 patients  Mean follow-up 8 months  Setting: 12 different studies | FEV_1_%: 44%. Range?  Comorbidity: 45% CVD | Thorough modeling of many **pragmatic** predictors to predict mortality, exacerbation and hospitalization, simple enough for primary care. | Pooled analysis of 12 RCT’s | Different cohort: Kaplan-Meier   - Mortality: ns - Hospital: p<0.001 - Exacerbation: not shown | Fairly poor | 2 | - | Exacerbation ^18^ |
| **COPDSS: COPD Severity Score**  2008 | 267 patients  0-2 years follow-up  Home setting, United States | Population-based  FEV_1_%: 54%. Range?  Sociodemographic diversity | Preliminary set model to stage severity for epidemiologic use. Now **pragmatic** risk-adjustment to identify high-risk patients to individually target care. | (validation derived from model) Prospective observational cohort | Validation by different time interval: similar results.  Prior study: concurrent validity. | Fair | 7 | - | - |
| **DOREMI BOX**  2008 | 84 patients (68 follow-up)  > 36 months follow-up  ?respiratory clinic?, Bulgaria | 100% male.  FEV_1_%: 35%, 18-73%  Co-morbidity excluded | Preliminary modified and simplification of BODE, to assess and stage COPD and improve death prediction. | Prospective observational cohort | Internal reliability consistency: correlation = 0.49 - 0.765. Concurrent valid: correl. = 0.52 | Fairly good | ? | - | - |
| **DOSE**  2009 | *Model*: 375 patients  *Validation*: 152 Holland, 460 UK (unknown follow-up), 81 Japan (3 months), 133 London (9 years).  Primary care. Japan resp. clinic | Diverse severity between studies: FEV_1_%: 42-67%. All <80% | Modeling of **pragmatic** items to easily stage severity in all settings and COPD grades. Next, to predict future events to guide management. | Cross-sectional (model)  Retrospective and prospective datasets (validation) | Each outcome by one of the validation cohorts (selective)  Concurrent validity for selective current disease states | Fairly poor | 3 | - | - |
| **HADO**  2006 | 611 patients. 3 years follow-up  Hospital affiliated outpatient clinics, Spain | 98% male (representative of Spain though), 48 packyears  FEV_1_%: 50%. All <80% | Preliminary set model to assess COPD for **pragmatic** and easy prediction of death at standard visit in daily practice. | Prospective observational cohort | Not shown. concurrent validity Implicitly with health related QOL. No validation group | Fairly good | 5 | - | - |
| **Niewoehner 1+2**  2007 | 1829 patients. 6 month follow-up  Veterans affairs Medical facilities, US | 99% male, (ex)smokers, 75% CVD. FEV_1_%: 36%. All <60%  Exclusion severe/unstable co-morbidities/CVD | Thorough modeling of many predictors to develop individual risk scores that predict exacerbation or hospitalization. | Multicenter randomized trial | No validation group  Internal validity by bootstraps:  Exacerbation c-index =0.66  Hospitalization c- index =0.73 | Fair | 27 | - | - |
| **PILE**  2010 | 268 patients  Mean follow-up 6.1 years  Community based, USA | 70-79 year “healthy subjects”  FEV_1_%: :63% Asthmatics? | Thorough modeling of some predictors for accurate prediction in elderly, to aid exploration of use in clinical practice. | Subanalysis of community-based prospective dataset | Internal validity by HR confidence intervals by bootstrapping | Fair | 0 | - | - |
| **SAFE**  2007 | 86 patients, 1 year follow-up and previous, resp. clinic, Malaysia | 88% male, (ex)smokers  FEV_1_%: 43%, 12-98% | Preliminary set **pragmatic** model for holistic staging severity in daily practice | Prospective observational cohort | Internal reliability consistency:  Correlation = 0.621 - 0.801 | Fair | 5 | - | - |
| **Schembri et al.**  **(TARDIS)** 2009 | 3343 patients, Median follow-up 1.9 years. GP’s, Scotland | Seems unselected and diverse  FEV_1_%::? All <80% | Thorough modeling of many predictors for accurate clinical utility | Prospective observational cohort | No validation group  Nothing else shown | Fairly poor/fair | 0 | - | - |

**# Poisson coefficient per point; ns: Not significant; SCI: Science Citation Index; Val. is validation**

|  | **Index information, predictors** | |  | **Index information, outcome** | |
| --- | --- | --- | --- | --- | --- |
| **Index** | **Predictors** | **FEV_1_ cut-points** | **Predictor significance** | **Prognostic outcome** | **Prognostic value and accuracy** |
| **ADO**  10-points scale | Age  Dyspnoea (MRC or GCRQ)  Obstruction (FEV_1_%) | <35; 35-50; 50-65; >65  (Spanish guides) | All significant in model building (multivariable logistic regression) | Any death | *Validation group*:  C-statistic = 0.63 (BODE 0.62)  OR 1.37/point  Hosmer-Lemeshow = 0.98 (BODE = 0.04) |
| **BODE**  10-point scale  4 quartiles | BMI (length/weight^2^)  Obstruction (FEV_1_%)  Dyspnoea (MRC score)  Exercise tolerance (6MWD) | <35; 35-50; 50-65; >65 (ATS 1991, incorrect) | All significant in model development by stepwise forward regression analyses | Any death  Respiratory death | *Validation group*:  C-statistic any death=0.74. (FEV_1_% =0.65)  Cox HR any death =1.34/point  Cox HR resp. death =1.62/point |
| - ***BODEx***   *9-point scale* | *BMI (length/weight^2^)*  *Obstruction (FEV_1_%)*  *Dyspnoea (MRC score)*  *Exacerbations* | *<35; 35-50; 50-65; >65*  *(as by BODE)* | *All significant except BMI* | *Any death* | *Cox HR 1.44/point*  *C-statistic =0.74*  *(BODE HR 1.33/point, c-statistic=0.75)* |
| - ***e-BODE***   *12-point scale* | *Exacerbations*  *BMI (length/weight^2^)*  *Obstruction (FEV_1_%)*  *Dyspnoea (MRC score)*  *Exercise tolerance (6MWD)* | *<35; 35-50; 50-65; >65*  *(as by BODE)* | *All significant except BMI* | *Any death* | *Cox HR 1.35/point*  *C-statistic =0.77*  *(BODE HR 1.33/point, c-statistic=0.75)* |
| - ***mBODE***   *10-point scale* | *BMI (length/weight^2^)*  *Obstruction (FEV_1_%)*  *Dyspnoea (MRC score)*  *Exercise max. O_2_ use* | *<35; 35-50; 50-65; >65 (ATS 1991, incorrect)* | *Only VO_2_:*  *Correlation VO2%: 0.64* | *Association with BODE* | *Correlation: Pearson’s r = 0.95* |
| **CPI: COPD Prognostic Index**  100-point scale  3 categories | Quality of life (SGRQ/CRQ)  Obstruction (FEV_1_%)  Age  Gender  BMI  History of ED/exacerbation  History of CVD | <30; 30-50; 50-60; >60  (data-dependent) | All significant for ≥ 1 outcome, by backward stepwise procedures. | Any Death  Any hospitalization  Exacerbation | *Model*:  C-statistic of both death and hospital=0.71  *Validation group*:  C-statistic not reported. HR increase 54%, 57% and 21% per 10 points resp. |
| **COPDSS: COPD Severity Score**  (questionnaire)  35-point scale | Respiratory symptoms  Systemic corticosteroids  Other COPD medications  Hospitalization/Intubation  Home Oxygen | - | Not shown | Respiratory outpatient  Respiratory ED visit  Respiratory hospital | *Validation group*:  Reflected by nomogram  Adjusted OR = 1.54-1.99 per 3 points |
| **DOREMI BOX**  10-point scale  2 categories | Dyspnoea (ATS)  Obstruction (FEV_1_%)  Rate of Exacerbation  Movement (6MWD)  BMI (length/weight^2^)  Blood OXygen (PaO_2_) | <50 ; 50-80 ; >80 (Adapted Gold) | All significant, though 6MWD and dyspnoea not shown | Any death  Association with BODE | Cox HR = 1.44 per point  (BODE Cox HR = 1.24 per point)  Correlation BODE: r = 0.87 |
| **DOSE**  8-point scale | Dyspnoea (MRC score)  Obstruction (FEV_1_%)  Smoking  Exacerbations | <30; 30-50; 50-80 (Gold) | Not shown | Japan: Assoc. BODE  London: exacerbation/ hospital for exac.  (retrosp/prosp)  UK: Beddays (retrosp) | Correlation BODE: Spearman’s r =0.78  Hospital: prosp.= ns#, ROC =0.76  Exacerbation: prosp. =1.07#  Beddays: Pearson’s r =0.33 |
| **HADO**  12-point scale  3 categories | Health (new questionnaire)  Activity (new questionnaire)  Dyspnoea (Fletcher)  Obstruction (FEV_1_%) | <35; 35-50; 50-65; >65 (ATS 1995, incorrect) | Modeled together components loose most significant effect on outcome | Any death | C-statistic =0.68. (FEV_1_% =0.65)  Adjusted OR = 0.18 for best category |
| **Niewoehner (1)**  422-point scale | Age  Obstruction (FEV_1_%)  Hospitalization  COPD duration  Productive cough  Antibiotics  Systemic corticosteroids  Theophylline | 10-19; 20-29; 30-39; 40-49; 50-59; 60-69  (data-dependent) | All significant in model development by stepwise regression analyses | Exacerbation | c-index = 0.67  risk nomogram  calibration plot appears good |
| **Niewoehner (2)**  249-point scale | Age  Obstruction (FEV_1_%)  Hospitalization  Unscheduled visits  Cardiovascular disease  Oral corticosteroids | 10-19; 20-29; 30-39; 40-49; 50-59; 60-69  (data-dependent) | All significant in model development by stepwise regression analyses | Hospitalization for exacerbation | c- index = 0.75  risk nomogram  calibration plot appears good |
| **PILE**  10 point scale | Obstruction (FEV_1_%)  Interleukin-6  Knee extensor strength | 30-50; 50-80; >80 (ATS) | All significant | Any death | c-stat= 0.71 (mBODE=0.64, FEV_1_%=0.63)  adjusted Cox HR = 1.30/point |
| **SAFE**  9-point scale  4 quartiles | SGRQ score (questionnaire)  Air-flow limitation (FEV_1_%)  Exercise tolerance (6MWD) | <30; 30-50; 50-80; >80 (Gold) | Not shown | Exacerbation | Pearson’s r = 0.50 |
| **Schembri et al.**  **(TARDIS)**  16-point scale | Age  BMI  Dyspnoea (MRC score)  Obstruction (FEV_1_%)  Hospitalization  Influenza vaccination | <30; 30-50; 50-80  (not determined) | Weibull proportial HR:  All significant | Hospitalization for COPD or respiratory death as 1 outcome | Weibull proportial HR (not shown)  Cumulative risk nomogram |

Reference List

(1) Esteban C, Quintana JM, Moraza J et al. BODE-Index vs HADO-score in chronic obstructive pulmonary disease: Which one to use in general practice? *BMC Med.* 2010;8:28.

(2) Puhan MA, Garcia-Aymerich J, Frey M et al. Expansion of the prognostic assessment of patients with chronic obstructive pulmonary disease: the updated BODE index and the ADO index. *Lancet.* 2009;374:704-711.

(3) de Torres JP, Cote CG, Lopez MV et al. Sex differences in mortality in patients with COPD. *Eur Respir J.* 2009;33:528-535.

(4) Karoli NA, Rebrov AP. [The BODE index as a predictor of unfavourable prognosis in chronic obstructive pulmonary disease (by the results of a prospective study)]. *Ter Arkh.* 2007;79:11-14.

(5) Imfeld S, Bloch KE, Weder W, Russi EW. The BODE index after lung volume reduction surgery correlates with survival. *Chest.* 2006;129:873-878.

(6) Martinez FJ, Foster G, Curtis JL et al. Predictors of mortality in patients with emphysema and severe airflow obstruction. *Am J Respir Crit Care Med.* 2006;173:1326-1334.

(7) Soler-Cataluna JJ, Martinez-Garcia MA, Sanchez LS, Tordera MP, Sanchez PR. Severe exacerbations and BODE index: two independent risk factors for death in male COPD patients. *Respir Med.* 2009;103:692-699.

(8) Casanova C, Cote C, de Torres JP et al. Inspiratory-to-total lung capacity ratio predicts mortality in patients with chronic obstructive pulmonary disease. *Am J Respir Crit Care Med.* 2005;171:591-597.

(9) Ong KC, Earnest A, Lu SJ. A multidimensional grading system (BODE index) as predictor of hospitalization for COPD. *Chest.* 2005;128:3810-3816.

(10) Lin YX, Xu WN, Liang LR et al. The cross-sectional and longitudinal association of the BODE index with quality of life in patients with chronic obstructive pulmonary disease. *Chin Med J (Engl ).* 2009;122:2939-2944.

(11) Faganello MM, Tanni SE, Sanchez FF, Pelegrino NR, Lucheta PA, Godoy I. BODE index and GOLD staging as predictors of 1-year exacerbation risk in chronic obstructive pulmonary disease. *Am J Med Sci.* 2010;339:10-14.

(12) Cote CG, Dordelly LJ, Celli BR. Impact of COPD exacerbations on patient-centered outcomes. *Chest.* 2007;131:696-704.

(13) Marin JM, Carrizo SJ, Casanova C et al. Prediction of risk of COPD exacerbations by the BODE index. *Respir Med.* 2009;103:373-378.

(14) Ko FW, Tam W, Tung AH et al. A longitudinal study of serial BODE indices in predicting mortality and readmissions for COPD. *Respir Med.* 2010.

(15) Martinez FJ, Han MK, Andrei AC et al. Longitudinal change in the BODE index predicts mortality in severe emphysema. *Am J Respir Crit Care Med.* 2008;178:491-499.

(16) Lopez-Campos JL, Cejudo P, Marquez E et al. Modified BODE indexes: Agreement between multidimensional prognostic systems based on oxygen uptake. *Int J Chron Obstruct Pulmon Dis.* 2010;5:133-140.

(17) Cote CG, Pinto-Plata VM, Marin JM, Nekach H, Dordelly LJ, Celli BR. The modified BODE index: validation with mortality in COPD. *Eur Respir J.* 2008;32:1269-1274.

(18) Eisner MD, Omachi TA, Katz PP, Yelin EH, Iribarren C, Blanc PD. Measurement of COPD severity using a survey-based score: validation in a clinically and physiologically characterized cohort. *Chest.* 2010;137:846-851.
